# Intensity modulated photocurrent spectroscopy to investigate hidden kinetics at hybrid perovskite–electrolyte interface

**DOI:** 10.1038/s41598-022-16353-6

**Published:** 2022-08-20

**Authors:** Priya Srivastava, Ramesh Kumar, Hemant Ronchiya, Monojit Bag

**Affiliations:** 1 Advanced Research in Electrochemical Impedance Spectroscopy Laboratory, Indian Institute of Technology Roorkee, Roorkee, 247667 India; 2 Centre for Nanotechnology, Indian Institute of Technology Roorkee, Roorkee, 247667 India

**Keywords:** Energy science and technology, Materials science, Physics

## Abstract

The numerous assorted accounts of the fundamental questions of ion migration in hybrid perovskites are making the picture further intricate. The review of photo-induced ion migration using small perturbation frequency domain techniques other than impedance spectroscopy is more crucial now. Herein, we probe into this by investigating perovskite–electrolyte (Pe–E) and polymer-aqueous electrolyte (Po–aqE) interface using intensity modulated photocurrent spectroscopy (IMPS) in addition to photoelectrochemical impedance spectroscopy (PEIS). We reported that the electronic-ionic interaction in hybrid perovskites including the low-frequency ion/charge transfer and recombination kinetics at the interface leads to the spiral feature in IMPS Nyquist plot of perovskite-based devices. This spiral trajectory for the perovskite-electrolyte interface depicts three distinct ion kinetics going on at the different time scales which can be more easily unveiled by IMPS rather than PEIS. Hence, IMPS is a promising alternative to PEIS. We used Peter’s method of interpretation of IMPS plot in photoelectrochemistry to estimate charge transfer efficiency $({Q}_{ste})$ from the Rate Constant Model. The ${Q}_{ste}$ at low-frequency for Pe–E interface exceeds unity due to ion migration induced modified potential across the perovskite active layer. Hence, ion migration and mixed electronic-ionic conductivity of hybrid perovskites are responsible for the extraordinary properties of this material.

## Introduction

Ion migration in hybrid organic–inorganic perovskite (HOIP) materials is still mysterious even after almost a decade of research. Leading to the photocurrent–voltage hysteresis, negative capacitance, reversible photodegradation of solar cells, instability to light, heat and moisture, photo-induced ion migration is challenging the application of perovskite-based devices in the commercial markets^1–4^. With diverse explanations to the basic questions such as which ions are migrating, what are the channels of ion migration, how the ion migration is affecting the device efficiency and stability and the methods to suppress ion migration in HOIP materials, the picture of ion migration in HOIPs is more intricate now^5–8^. Even though there have been many attempts to investigate ion migration under different operating conditions^9,10^, the complete picture is still not clear. In the last decade, most of the conventional studies on the fundamentals of ion migration were performed on the solid-state device geometries^3,10–12^. In 2015, Bag et al. demonstrated the quasi-reversible photodegradation in p–i–n perovskite device architecture^3^. They claimed that operational lifetime and stability of these devices can be improved with cooling or with infrared cutoff filter. About a year later, reports on slow photodegradation due to the formation of light activated meta-stable deep-level trap state and the self-healing of perovskite solar cells by resting them in dark or operating at 0 °C was published^12^. Following that there have been many reports on the nature of ionic defects^13^, activation energy of ion conduction^14^, interplay between electronic and ionic charge carriers^15^, channels of ion migration^8^, light-soaking effects and minimizing hysteresis^16^.

Most of these fundamental studies on ion migration in HOIP materials have been performed on solid-state device geometry. Lateral device geometry (Au/perovskite/Au)^11^, conventional solid-state p–i–n and n–i–p perovskite solar cells^17,18^, and single crystals^19,20^ are some of the mostly used device architectures. In case of conventional perovskite solar cell geometry, the selective charge transport layers are also used. Smith et al. determined that ions respond to bias and illumination in a predictable manner in solid-state HOIP photovoltaic devices^9^. This migration of ions is highly influenced by the energy levels (HOMO and LUMO) of charge selective transport layers as the specific ion migrates through the bulk and accumulate at specific interface. Moreover, in solid-state device geometries the electronic current predominates ionic current and the ion kinetics in the perovskite bulk is highly influenced by this^21^. In that case, the controlled ion migration is studied which fails to decipher the ion kinetics and transport in the unrestrained environment. However, in case of solid–liquid junction-based devices the redox potential of the liquid electrolyte acts as a buffer energy level which suppresses the electronic contribution of the current while measuring the frequency-dependent ion kinetics in HOIP active layer. Therefore, ion transport can be examined more explicitly in this type of device geometry^21,22^. Recently, Kamat group have started investigating charge and mass (ion) transfer using solid–liquid interface architecture. Many reports on the role of A-site cation in dictating mobility of halide ions^23^, suppression of electrochemically induced iodine migration through chloride insertion^24^ and modulation of photoinduced iodide expulsion in HOIPs through electrochemical bias^25^ are performed on the perovskite–electrolyte interface-based devices. We have also investigated the ionic-electronic interaction under different operating conditions leading to tunable ambipolarity of perovskites^26^ and tuning the ionic conductivity by modulating the A-site cation and X-site halide ions in the perovskites^27^ using a similar device architecture. Moreover, understanding the photo physics at perovskite–electrolyte interface can be useful in the application of hybrid perovskites in photo-rechargeable electrochemical cells^28^, electrolyte gated field effect transistors (FETs)^29^, photoelectrochemical (PEC) and photocatalytic (PC) cells for water-splitting^30^, supercapacitors^28,31^ and CO_2_ capture and reduction devices^32^. However, the precise interplay between ion movement and electronic kinetics in these devices presents a challenge for optimizing these devices. In recent work, our group has demonstrated that ion migration in perovskites and liquid electrolytes plays a significant role in the electrostatic double layer -and diffusion-limited capacitances^31^.

In addition, the instability of hybrid perovskite materials in contact with aqueous electrolyte is complicating the research and application in this direction. There are two ways to address this issue. Either hybrid perovskite film in contact with non-aqueous solvent can be inspected as performed in previous studies or a protective polymer film can be introduced between the hybrid perovskite thin film and aqueous electrolyte. To explore these two options, we need to investigate the difference between the charge and ion kinetics at perovskite/non-aqueous interface and polymer/aqueous interface. Here, we are presenting a comparative investigation of the perovskite/non-aqueous electrolyte interface with polymer/aqueous electrolyte interface using small perturbation techniques.

In addition to the use of photoelectrochemical impedance spectroscopy (PEIS) there have been several reports on intensity modulated photocurrent/photovoltage spectroscopy (IMPS/IMVS) on perovskite based solid state devices, mainly for the solar cells and light emitting device applications^33–38^. Although PEIS has already been used to study semiconductor/electrolyte interface before^39^, the use of IMPS/IMVS is still lacking in the literature. This could be due to weak steady state current in some semiconductor/electrolyte-based device or due to the complexity of the system itself. Here, we examine the charge kinetics and ion transport at the perovskite–electrolyte interface in more detail by comparing the device behavior with polymer–electrolyte interface. We have carried out the PEIS and IMPS measurements of both devices to interpret carrier recombination times, electro-ionic dynamic at the interface, and ionic conductivity. For the perovskite–electrolyte based devices, formamidinium lead iodide (FAPbI_3_) thin perovskite films in contact with 0.1 M tetrabutylammonium perchlorate (TBAClO_4_) in dichloromethane (DCM) is used. For the polymer-electrolyte -based device a thin film of poly-3-hexylthiophene (P3HT) in contact with 0.1 M aqueous solution of potassium chloride (KCl) is used. It can be noted that the interplay between electronic and ionic charge carriers and mixed electronic-ionic conduction in HOIPs is responsible for the extraordinary properties of this material particularly at mid- and low frequencies.

## Results

### Small perturbations techniques for HOIPs

Frequency domain small perturbation modulation techniques such as impedance spectroscopy (IS), intensity modulated photocurrent spectroscopy (IMPS), and intensity modulated photovoltage spectroscopy (IMVS) are powerful tools to characterize solid-state light-to-energy conversion photovoltaic and photoelectrochemical devices under operating conditions^36,40–44^. The optoelectronic device is perturbed by a small electrical signal in IS, whereas IMPS/IMVS involves optical perturbation in incident illumination. IS interprets the information of both charge carrier transport and recombination processes together occurring in the device. However, IMPS and IMVS individually study the charge carrier transport and recombination kinetics, respectively.

In IS, a small AC perturbation in voltage ($\widetilde{{V}_{e}}$) is applied and the corresponding AC current $\widetilde{{j}_{e}}$ is recorded over a range of frequency. The IS transfer function is generally represented as

1 $$\begin{array}{c}Z=\frac{\widetilde{{V}_{e}}(\omega )}{\widetilde{{j}_{e}}(\omega )}.\end{array}$$

For IMPS and IMVS measurements, a perturbation of incident photon current density ($\widetilde{{j}_{\varphi }}=e\widetilde{\varphi }$) is applied and the corresponding photocurrent ($\widetilde{{j}_{e}}$) at short-circuit (i.e., $V=0$) condition and photovoltage ($\widetilde{{V}_{e}}$) at open-circuit condition (i.e., $J=0$) over the entire frequency range is measured, respectively. The IMPS and IMVS transfer functions can be represented as

2 $$\begin{array}{c}Q=-\frac{\widetilde{{j}_{e}}\left(\omega \right)}{\widetilde{{j}_{\varphi }}\left(\omega \right)},\end{array}$$

3 $$\begin{array}{c}W=\frac{\widetilde{{V}_{e}}(\omega )}{\widetilde{{j}_{\varphi }}(\omega )}.\end{array}$$

It is to be noted that one of the quantities, either $\widetilde{{j}_{e}}(\omega )$ or $\widetilde{{V}_{e}}(\omega )$ should be negative. These three techniques (IS, IMPS, and IMVS) are closely related to each other. The basic relationship between the transfer functions Z, Q, and W is^45^

4 $$\begin{array}{c}Z\left(\omega \right)=\frac{W\left(\omega \right)}{Q\left(\omega \right)}.\end{array}$$

The data is mostly represented in terms of Nyquist plot which is the variation of imaginary part with the real part [i.e., $- Z^{\prime\prime}\left( \omega \right)$ with $Z^{\prime}\left( \omega \right)$ for IS, $- Q^{\prime\prime}\left( \omega \right)$ with $Q^{\prime}\left( \omega \right)$ for IMPS or $- W^{\prime\prime}\left( \omega \right)$ with $W^{\prime}\left( \omega \right)$ for IMVS]. The other form of representation in the bode plot which is plot of magnitude of impedance/photocurrent/photovoltage, and phase versus frequency. Small perturbation techniques have been instrumental in characterization of electronic and ionic behaviour in HOIP materials. On one hand where IS has proved to be helpful technique to study kinetics of ion transport in perovskites^3,9^, IMPS/IMVS emerged as advantages tool to explore the frequency domain properties of the perovskite devices^36,46^. The low-frequency (LF) capacitance which is the characteristic feature of the ordinary HOIP configuration is directly or indirectly responsible for a range of steady-state and dynamic effects in perovskite photovoltaic devices. This LF capacitance is generated by the accumulation of ionic and electronic charge carriers at the perovskite/selective contact interface^46^. These interfaces with sharp potential drops serve as preferential regions for recombination and affects the charge extraction significantly^47^. These effects are directly connected to the mechanism of degradation in HOIPs and govern the stability and performance of the PSCs. Impedance spectroscopy (IS) have been extensively applied in perovskite solar cell research to characterize the physical processes in photovoltaic devices^3^. However, the interpretation of IS Nyquist plot has been complex and is still debatable. Moreover, some mid-frequency features are not clearly unveiled by IS measurements in hybrid perovskite devices^48^. IMPS technique have already been extensively applied to decouple charge transfer and recombination kinetics at semiconductor–electrolyte interface^49,50^ and study the transport and recombination processes in dye-sensitized solar cells (DSSCs)^51^, photoelectrodes for water splitting^43^, and recently they are gaining tremendous attention in field of charge and ion studies in hybrid perovskite materials^35,41^. Therefore, exploring the charge and ion kinetics in HOIP materials using IMPS technique in addition to IS will enable to overcome their individual limitations and extract maximum information from the recorded results. Investigation of the LF phenomenon in HOIP devices using frequency domain small perturbation techniques other than IS is crucial for further improvement in stability and efficiency.

### Intensity modulated photocurrent spectroscopy

**Figure 1:**
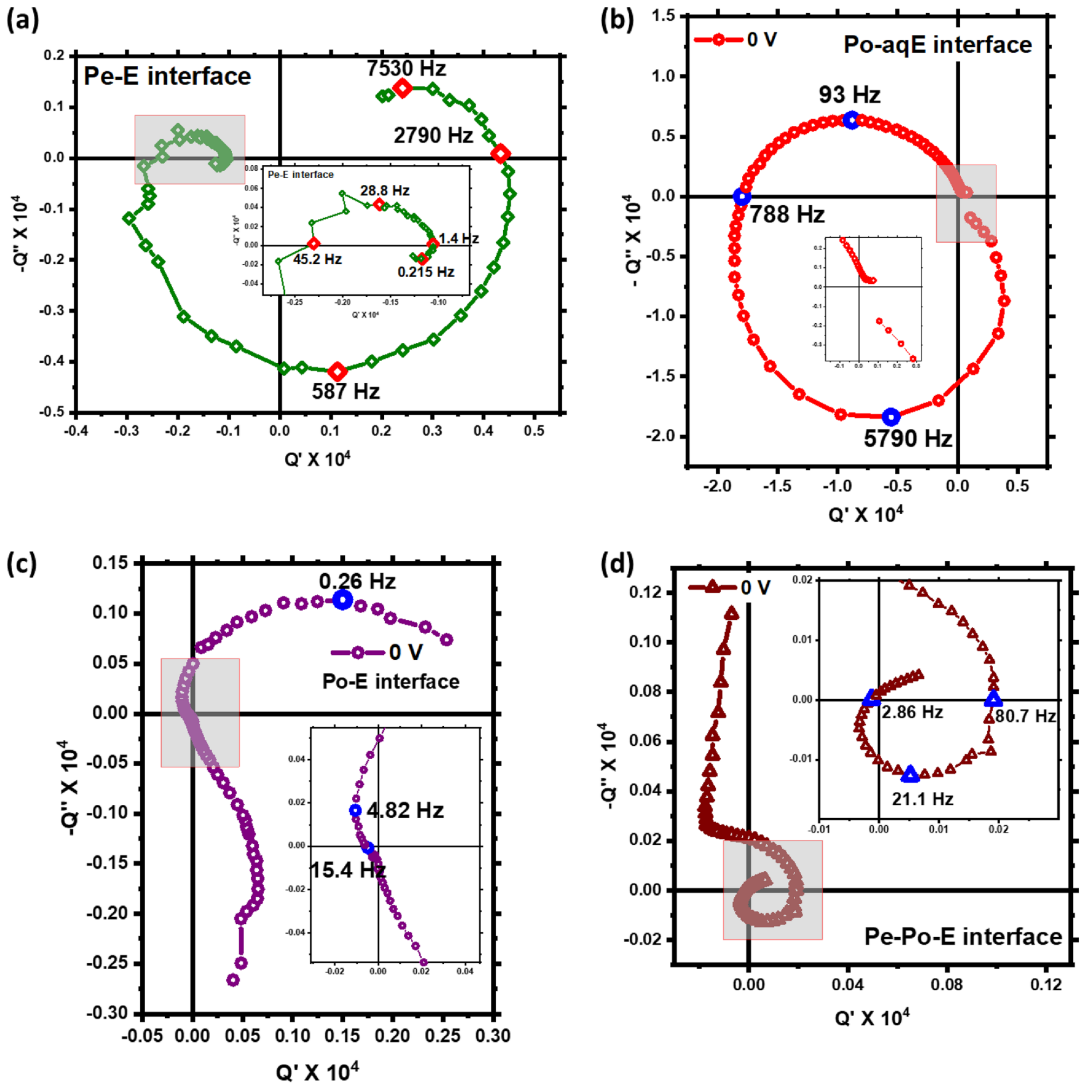
IMPS Nyquist plot of (**a**) Pe–E (perovskite–electrolyte interface), (**b**) Po–aqE (polymer–aqueous electrolyte interface), (**c**) Po–E (polymer–electrolyte interface) and (**d**) Pe–Po–E (perovskite–polymer–electrolyte interface).

The IMPS measurements were performed at the perovskite–electrolyte (ITO|PEDOT: PSS|FAPbI_3_| TBAClO_4_ in DCM| Pt) and polymer-aqueous electrolyte (ITO|P3HT| aqueous solution of KCl| Pt) interface. The complex plane plot with the real and imaginary photocurrent transfer function is shown in the Fig. 1. Considering the holes are collected at the ITO|PEDOT: PSS contact and electrons at Pt electrode, the positive real photocurrent is cathodic and vice-versa. It can be noted that, the P3HT-aqueous electrolyte system operates at mostly in anodic direction whereas the perovskite–electrolyte system sweeps all four quadrants.

According to the literature, IMPS response generally depicts two main mechanisms: the relaxation of the transported photogenerated charge carriers on discrete and distributed surface states and slow transport of photogenerated charge carriers to the interface with or without recombination and trapping in the bulk^52–54^. In the first mechanism, the carriers are rapidly transported to the interface and charge it. As the charge carriers increase at the surface states, they start recombining. In this process, the photocurrent leads the light intensity in-phase and the photocurrent magnitude increases with increase in modulation frequency. In the second process, the charge carriers are slow and there is no sudden spike when the light is turned on since the charge carriers take time to reach the interface. In this case, the photocurrent lag behind the light intensity and hence the photocurrent has negative phase. Moreover, the photocurrent magnitude decreases with increase in the light modulation frequency approaching zero at higher frequencies since the charge carriers do not have enough time to reach the interface. At higher frequency, the transport limited photocurrent corresponds to the IMPS plot in IVth quadrant (or II quadrant in case of anodic operation mode). However, transportation is no longer an issue at lower frequencies and the relaxation at surface states play an important part. This corresponds to the IMPS plot in Ist quadrant (or IIIrd quadrant in anodic mode).

**Figure 2:**
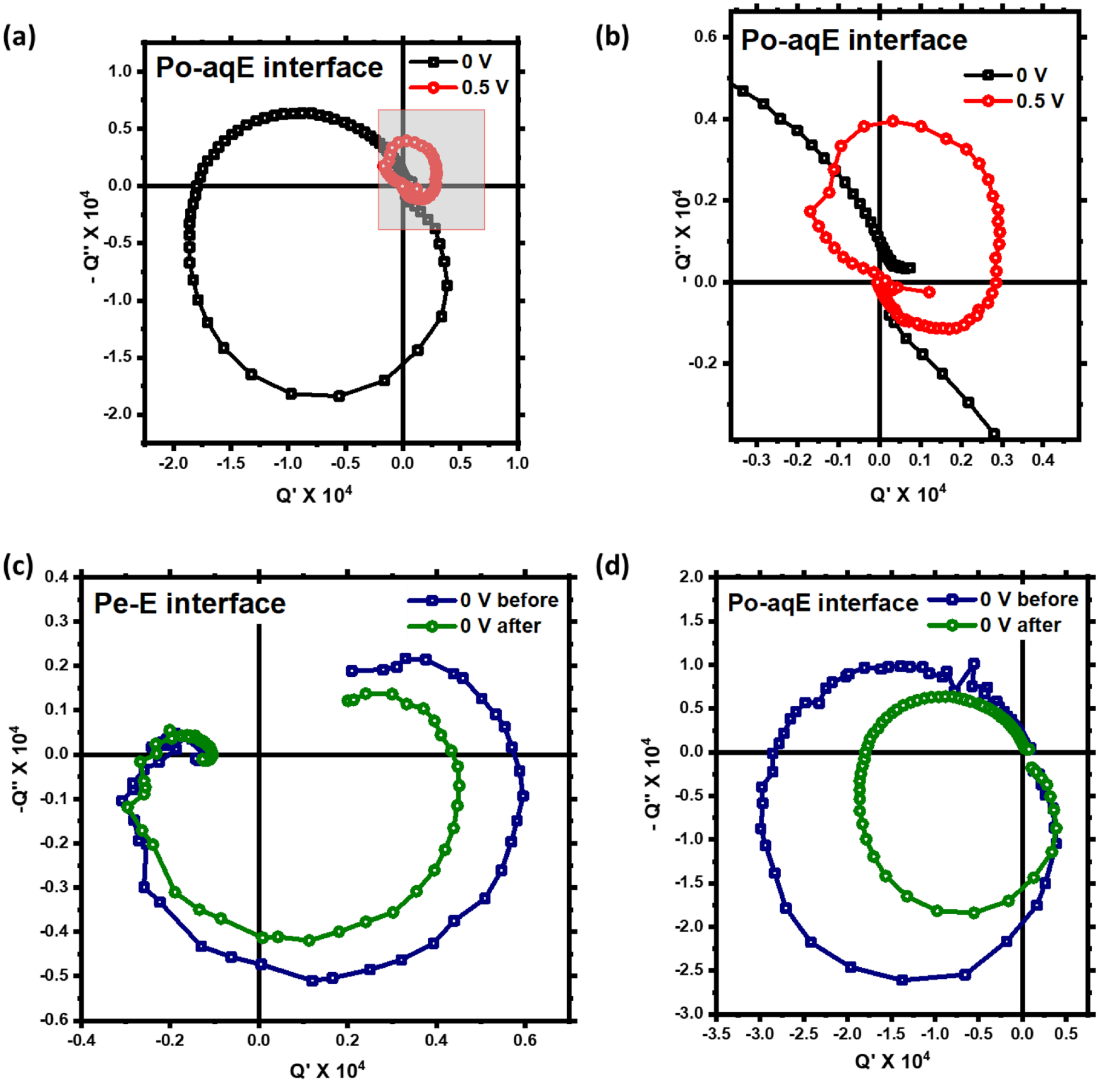
(**a**,**b**) IMPS transfer function for Po–aqE interface at 0 V and 0.5 V applied bias, (**c**,**d**) IMPS Nyquist plot before and after exposure to illumination for Pe–E and Po–aqE interface, respectively.

**Figure 3:**
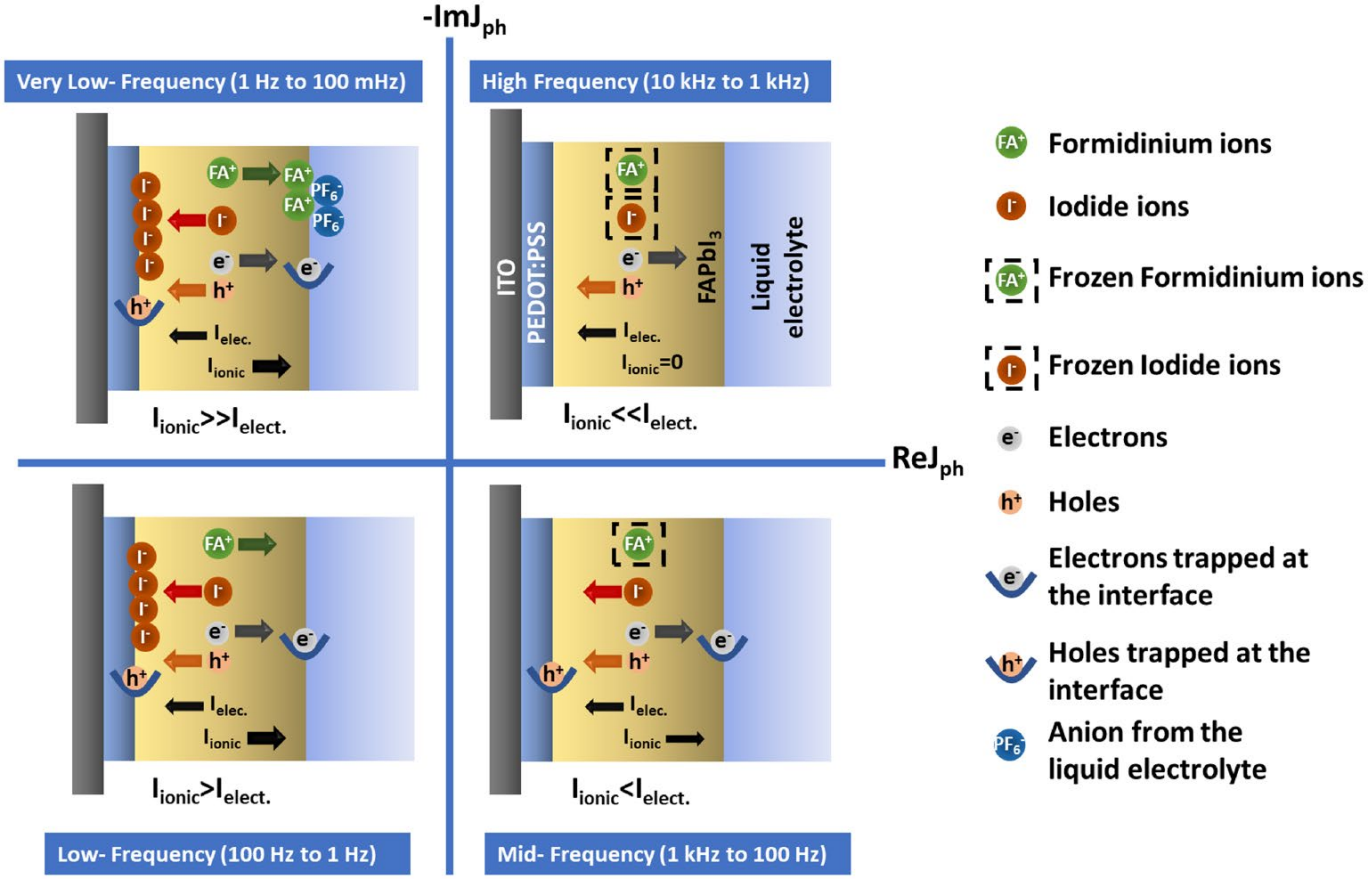
Schematics of electronic-ionic kinetics at different frequency leading to different feature in the four quadrants of Q-plane plot of perovskite–electrolyte interface.

The IMPS plot (Fig. 1a) we obtained for Pe–E from the experiments in this study sweeps through almost all four quadrants. This depicts the presence of two photocurrent origins with opposite sign (cathodic and anodic) and different characteristic relaxation times. The origin of photogenerated anodic current can be attributed to the movement of photoexcited electrons and holes towards ITO|PEDOT: PSS or ITO|P3HT and Pt electrodes, respectively. It is reported earlier that the built-in field fails to guide the photogenerated holes to ITO|PEDOT: PSS or ITO|P3HT electrode due to pronounced fermi-level pinning and the photoexcited charge carriers are transported by means of diffusion^21,52^. In the short-circuit condition, this anodic current is dominating over the cathodic in P3HT-aqueous electrolyte devices. However, on application of applied bias the current is no more diffusion limited and the device shows the cathodic behavior. This can be seen by complete flipping of the IMPS Nyquist plot for P3HT-aqueous electrolyte interface in Fig. 2a,b. Although, in case of perovskite–electrolyte device both anodic and cathodic currents are comparable. This can be attributed to the diffusion limited electronic and ionic current in the perovskite material where ionic current can be observed at low-frequency leading to anodic feature. There is a possibility that the built-in and photogenerated field is driving the current leading to drift-limited current even in the short circuit condition^9,26^. The change in the sign of the photocurrent can be understood with the help of a schematic diagram (Fig. 3) based on the electron–ion interaction. The change of current is not because of the anodic or cathodic nature of the interface but due to a strong or weak ionic interaction at different modulation frequencies^55^. The net current is the result of photogenerated electronic current as well as the ionic contribution to the particular frequency. The opposite nature of electronic and ionic current leads to the flipping of current at certain frequencies.

**Figure 4:**
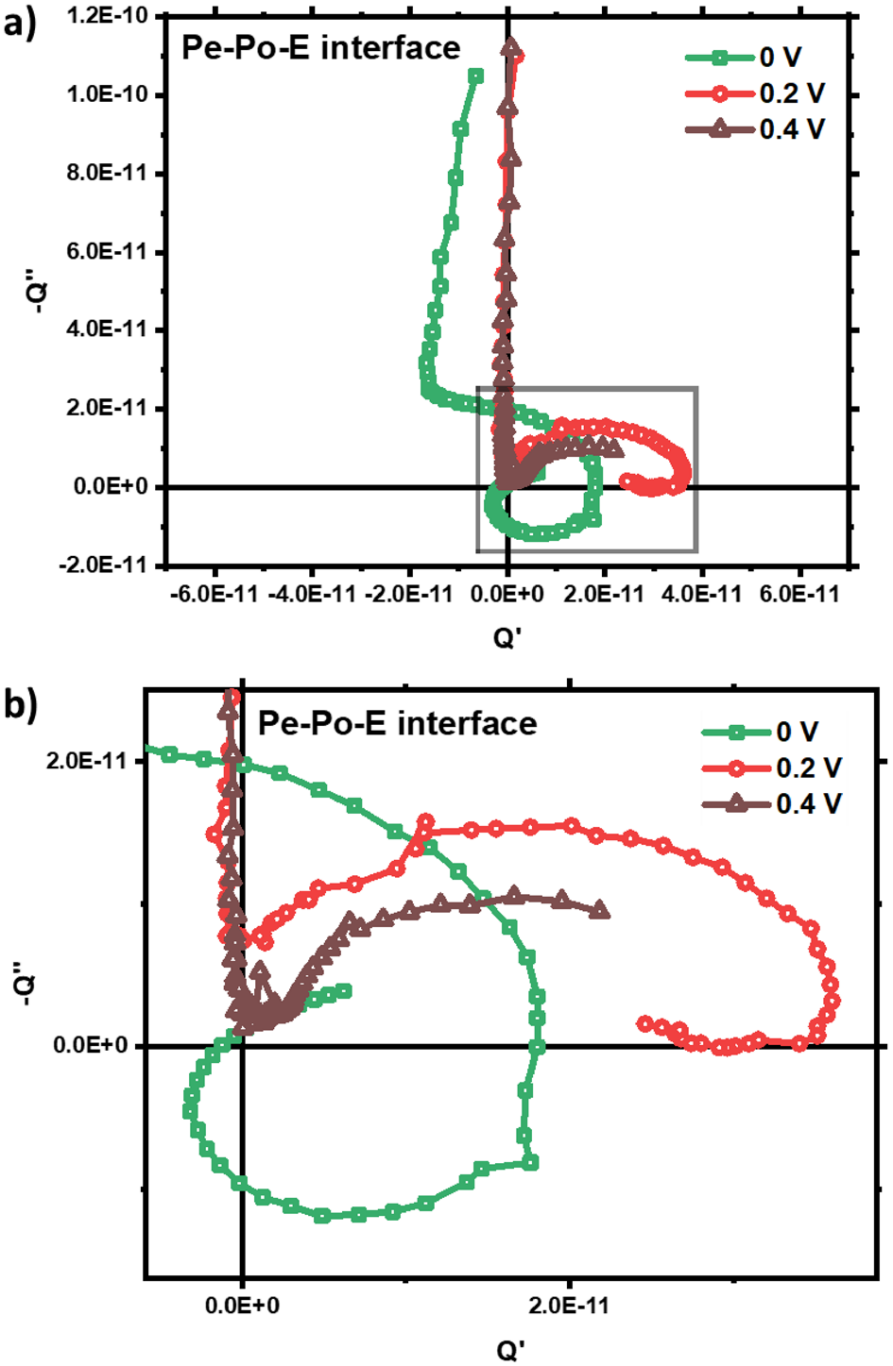
(**a**) IMPS Spectra of perovskite–polymer–electrolyte interface device at different applied bias (0, 0.2, 0.4 V), (**b**) zoomed view of the corresponding low-frequency feature.

Moreover, we have performed the IMPS measurements on perovskite–polymer–electrolyte (Pe–Po–E interface) device geometry at different applied bias 0, 0.2, 0.4 V. Applied bias changes the kinetics at the interface, therefore the shape of the curve is significantly different from the zero-bias condition as seen from Fig. 4a,b. More analysis is needed to understand the bias dependent IMPS in perovskite/electrolyte interface. We have inspected the effect of photodegradation on the IMPS feature for Pe–E and Po–aqE interfacial device by recording the IMPS data of the devices before and after exposure to illumination (Fig. 2c,d). It can be observed that the nature of the Q-plane plot remains same after photo illumination. However, the magnitude of photocurrent is reduced which can be attributed to the trapping of electronic charge carriers at the vacancies and defects in the perovskite bulk and at the interfaces.

**Figure 5:**
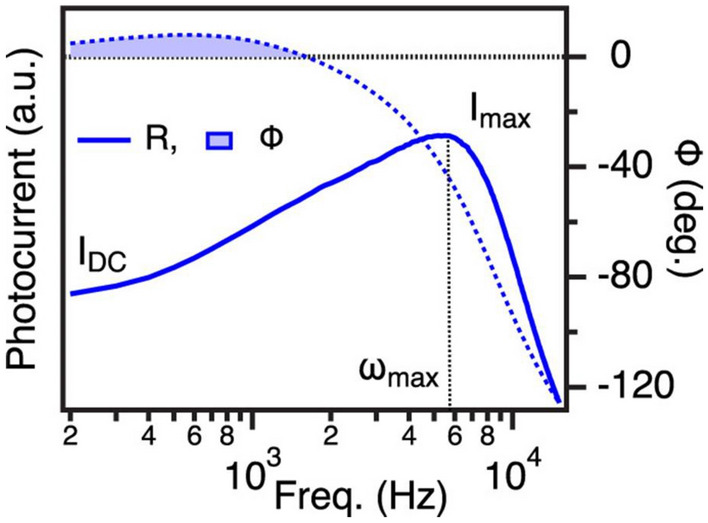
Typical IMPS response characteristics of an organic solar cell showing light degradation. Photocurrent magnitude (R) and phase shift (ϕ) are displayed in the Bode representation. Reprinted with permission from Ref.^57^. Copyright 2020 American Chemical Society.

**Figure 6:**
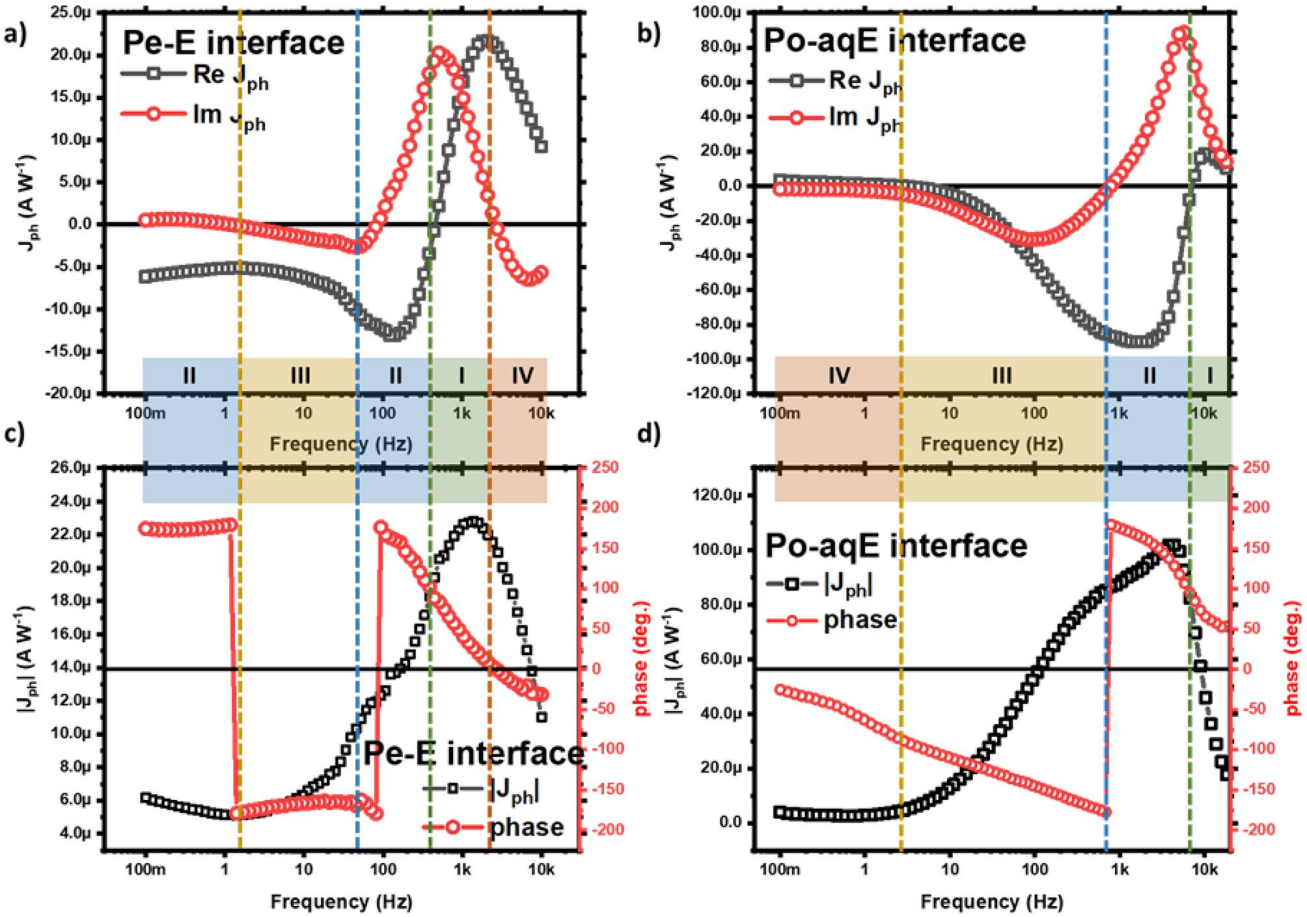
Real and imaginary photocurrent versus frequency plot and Bode (|Z| and phase shift vs. frequency) plots for perovskite–electrolyte (**a**,**c**) and P3HT-aqueous electrolyte (**b**,**d**) interfaces.

Typical IMPS response for the solid-state P3HT: PCBM organic solar cell consists of photocurrent magnitude increasing with modulation frequency from steady-state dc levels featuring a gain peak (I_max_) at frequencies usually > 1 kHz accompanied by a rapid fall after ω_max_ (Fig. 5)^56,57^. The ratio I_max_/I_DC_ implies the effect of trap assisted recombination, charge traps and occupancies and varies with device operating condition, active layer morphology and aging. The phase shift is positive at low-frequency and gradually turns to negative at higher frequency regime. However, the IMPS response of P3HT-aqueous electrolyte device is little more complex than the solid-state devices (Fig. 6b,d). The photocurrent magnitude shows similar behavior of increasing with modulation frequency followed by a gain peak around 3–4 kHz. But the phase shift is positive at higher frequency flipping the polarity suddenly at around 1 kHz. Similar behavior is observed earlier by Semenikhin et al. in the study of polymer-based solar cell^52^. But the phase shift turns back to positive gradually towards low frequency. In Po–aqE devices studied here, the phase shift approaches zero but fails to turn positive till 100 mHz modulation frequency as evident from Fig. 6d.

The positive phase shift at high frequency (as photocurrent leads light intensity) for Po–aqE suggests that the observed recombination at high frequency is non-geminate or bimolecular recombination^58^. The photogenerated charge carriers separate and charge the interface as revealed by the growth of photocurrent magnitude with decrease in frequency in the HF regime. In the low-frequency regime (< 1 kHz), the IMPS response is largely determined by carrier transport. At low frequency, the interfacial recombination involving carriers that are already separated and trapped at the surface/interfacial states occurs. The negative phase shift at low-frequency suggests geminate or monomolecular recombination. In case of Pe–E device, the kinetics is much more complex. Although the magnitude of photocurrent pretty much follows the same trend with gain peak at high frequency, the phase shift switches more often as shown in Fig. 6c. For Pe–E interface the charge carriers follow almost similar kinetics to that of Po–aqE interface in the HF regime with positive phase shift suggesting non-geminate or bimolecular recombination. However, the low-frequency phase shift feature is different for Pe–E interface. The positive and almost constant phase shift at lower frequency flips to negative around 1 Hz modulation frequency. The magnitude of phase shift doesn’t change till around 100 Hz after which it again flips to positive value. There after it decreases gradually with modulation frequency turning negative around 2 kHz. This frequent flipping of the phase shift at LF regime can be attributed to the multiple ionic and electronic charge kinetics due to the accumulation and recombination of charge carriers at the perovskite/HTL and perovskite/electrolyte interface. The positive phase shift at low frequency can be attributed to the bimolecular recombination in hybrid perovskites due mixed electronic-ionic conduction in these materials^59,60^.

The real (ReJ_ph_) and imaginary (ImJ_ph_) photocurrent are negligible at very low frequency and shows similar trend. Both ReJ_ph_ and ImJ_ph_ first decreases (i.e., tends negative) with modulation frequency showing minima at intermediate frequency followed by a positive peak at higher frequency. Although the position and magnitude of these minima/maxima are different. The magnitude of anodic ReJ_ph_ maxima is approximately 4 times higher than that of cathodic peak for Po–aqE. For ImJ_ph_, the case is completely reversed. In case of Pe–E interface the ReJ_ph_ and ImJ_ph_ also display almost similar behavior to that of Po–aqE system (Fig. 6a). However, both the cathodic and anodic peaks of ReJ_ph_ are comparable in this case. The frequency at LF minima of ImJ_ph_ can be related to the rate of decay of photogenerated minority charge carriers at the surface states and it can be considered equal to the pseudo-first-order rate constant of the interfacial recombination $k$ in the first approximation, assuming no carrier extraction through the interfacial states^49,61,62^. The frequency at HF maxima of ImJ_ph_ can attributed to the bulk recombination indicating shorter carrier lifetime^58^. From Fig. 6a,b, it can be observed that the both type of recombination are higher for Po–aqE interface than Pe–E interface. This could be due to the presence of charge selective layer (PEDOT: PSS) in Pe–E device geometry.

We have also performed the IMPS measurements at the polymer–electrolyte (Po–E) interface with structure ITO|P3HT| TBAClO_4_ in DCM| Pt for non-aqueous electrolyte to elucidate the effect of nature of liquid electrolyte. It can be noted from Fig. 1c, that simple circular arc with no spiral feature at low frequency is observed. Hence, it can be interpretated that the liquid electrolyte just changes the mode of operation from anodic to cathodic depending upon the relative redox potential of the electrolyte with respect to the fermi level of the semiconductor. Though, no spiral feature at low frequency is observed for polymer–electrolyte interface. However, the IMPS spectra for perovskite-polymer integrated device geometry (Pe–Po–E) with structure ITO|FAPbI_3_|P3HT|TBAClO_4_ in DCM| Pt shows the spiral feature at low frequency (Fig. 1d). This proves that the mid- to low-frequency spiral feature in IMPS Q-plane plot is due to the multiple ionic and electronic charge transport and recombination kinetics at the perovskite bulk or interfaces with selective charge transport layers. The presence of the spiral feature in both perovskite/electrolyte and perovskite/P3HT/electrolyte interface reveals that this feature is due to the electronic-ionic kinetics in the perovskite bulk rather than at the interface.

On the basis of two capacitor approach by Wilson et al.^63^ Ponomarev and Peter provided understanding of IMPS feature of semiconductor-electrolyte interface through a fundamental model^50^. This model describes the competition between the transfer of minority charge carrier from the semiconductor thin film to the liquid-electrolyte through the interface at a characteristic rate of ${k}_{ct}$ and recombination with electrons in the conduction band at the semiconductor-electrolyte interface at a characteristic rate ${k}_{rec}$. Assuming a basic charge compensation between the space charge capacitance C_sc_ on the semiconductor side and the Helmholtz capacitance C_H_ on the electrolyte side at the interface, the IMPS transfer function $Q(\omega )$ can be obtained as

6 $$\begin{array}{c}Q\left(\omega \right)={Q}_{ste}\frac{1+i{\gamma }_{C}\omega {\tau }_{C}}{\left(1+i\omega {\tau }_{S}\right)\left(1+i\omega {\tau }_{t}\right)},\end{array}$$

where ${\gamma }_{C}=\frac{{C}_{tot}}{{C}_{SC}}$, and ${C}_{tot}=\frac{{C}_{SC}{C}_{H}}{{C}_{SC}+{C}_{H}}$. The surface charge transfer efficiency ${Q}_{ste}$, is an important quantity for the characterization of semiconductor-electrolyte interface. This can be expressed in terms of rate constant for charge transfer across the interface ${k}_{ct}$ and rate constant for charge recombination at the surface ${k}_{rec}$ as:

7 $$\begin{array}{c}{Q}_{ste}=\frac{{k}_{ct}}{{k}_{ct}+{k}_{rec}},\end{array}$$

where ${k}_{ct}$ and ${k}_{rec}$ can be related to charge transfer and recombination time constant as

8 $$\begin{array}{c}{\tau }_{C}=\frac{1}{{k}_{ct}},\end{array}$$

and

9 $$\begin{array}{c}{\tau }_{rec}=\frac{1}{{k}_{rec}},\end{array}$$

10 $$\begin{array}{c}{\tau }_{t}=\frac{1}{{k}_{ct}+{k}_{rec}}.\end{array}$$

Another time constant ${\tau }_{S}$, is defined in terms of series resistance R_S_ and total capacitance (C_tot_) as

11 $$\begin{array}{c}{\tau }_{S}={R}_{S}{C}_{tot}.\end{array}$$

Mostly the IMPS transfer function, exhibits two semicircles in the positive real half of the Nyquist plot. One with negative imaginary part and another with positive imaginary part. Here we will use Rate Constant Model (RCM) for IMPS analysis. This model has been extensively used for IMPS analysis in hematite photoanode studies^64–66^. The RCM model can be used to quantify ${Q}_{ste}$, ${k}_{ct}$ and ${k}_{rec}$ parameters. When the transfer function is normalized, the low frequency intercept on real axis directly represents charge transfer efficiency, otherwise it is the ratio of low frequency intercept to high frequency intercept. The maxima of the high frequency arc corresponds to ${\tau }_{S}$, whereas the low frequency arc maxima gives ${\tau }_{t}$^36,42^. We have extracted these parameters for both Pe–E and Po–aqE interfaces as listed in Table 1. In case of Pe–E interface we observed four semi-circles instead of two forming a spiral kind of trajectory. We can attribute the mid- and low-frequency spiral to the transport and recombination processes due to the ions in the perovskite and liquid electrolyte at the interface. The high frequency spiral is whereas due to the electronic charge transport and recombination, present in both Pe–E and Po–aqE interface. In this way, we can easily distinguish between the different processes occurring at different time scale in the device using IMPS which was otherwise difficult to elucidate from PEIS due to overlapping of the time constants leading to single semi-circle in the Nyquist plot. Hence, IMPS is a promising alternative technique to PEIS to explore the ion and charge kinetics in hybrid perovskites.

**Table 1:** The charge transfer efficiency (Q_ste_), charge transfer (*k*_ct_) and recombination (*k*_rec_) rate calculated from IMPS curve for Pe–E and Po–aqE interfaces.

Parameters	${Q}_{ste}$	${k}_{ct}$ (s^−1^)	${k}_{rec}$ (s^−1^)
**(1) Pe–E**			
(a) High-frequency	− 0.53	− 312.11	899.11
(b) Mid-frequency	0.46	13.21	15.59
(c) Low frequency	1.26	0.27	− 0.06
**(2) Po–aqE**			
(a) High-frequency	− 0.06	− 5.18	98.18

In the study of IS and IMPS spectra of PSCs by Ravishankar et al., it was observed that the IMPS Nyquist plot shows the existence of an additional characteristic process at intermediate frequencies (IF) in form of second arc in the upper quadrant for PSCs^48^. This feature was hidden in IS plots of the same devices. This feature was attributed to the accumulation of anions and electrons at the perovskite/HTL interface, considering the large value of the IF capacitance. Here, a similar IF arc can be noted for Pe–E and Pe–Po–E interfacial device from Fig. 1a,d, respectively. The accumulation of halide anions and electrons at perovskite/PEDOT: PSS and perovskite/P3HT interface might be responsible for this feature as suggested by Ravishankar et al. IMPS measurements are able to resolve feature like this at IF unlike IS in mixed ionic-electronic conductors such as HOIPs. Moreover, the low frequency capacitance and resistance is associated to the accumulation of ionic and electronic carriers at perovskite/ETL interface^67^ and their subsequent recombination^68^. In the liquid electrolyte-based device geometry studied here, the LF arc can be attributed to the accumulation and subsequent recombination of ionic and electronic charge carriers at perovskite–electrolyte in Pe–E and perovskite–ITO interface in Pe–Po–E device. The redox reactions occurring at the solid–liquid interface also affect the very low frequency kinetics and IMPS feature. Finally, it can be concluded that the mid- to low-frequency spiral observed in the IMPS Nyquist plot for perovskite-based devices with perovskite/HTL, perovskite/ETL or perovskite/electrolyte interfaces is due to the multiple ionic and electronic transport and recombination charge kinetics in the bulk and at interfaces.

The Nyquist plots of the transfer function reveal the interplay of three different processes going on at different time scale in Pe–E device. The negative sign in high-frequency charge transfer efficiency and charge transfer time constant is due to the transition from cathodic to anodic current direction or vice-versa. This feature of IMPS response function tending to the negative real part of the complex plane representation at low frequency leading to negative charge transfer efficiency has been reported earlier. This phenomenon usually occurs at the region of applied bias close to open circuit potential. It is mostly accompanied by the photocurrent sign switching, observed in linear sweep voltammetry in gold-decorated cadmium chalcogenide nanorods^69^, inject-printed CuBi_2_O_4_ photocathodes^70^, and in BiVO_4_ photoanodes^71^. While the switching of the IMPS response value at low frequency Q(0), to the negative values under different conditions, has been mostly associated with the change in the sign of the photocurrent in many reports, Morcoso et al. has shown that such interpretation is inconsistent with the experimental data measurements^33^. They demonstrated that the negative real low frequency IMPS response is not a consequence of photocurrent sign switching but the change in the extracted photocurrent with a change in the incident photon flux intensity or the differential external quantum efficiency (EQE_diff_) for solar energy conversion devices. The higher value of ${Q}_{ste}$ for Pe–E than Po–aqE reveals faster and efficient transfer of charge to the liquid electrolyte at perovskite–electrolyte interface. This could be due to large charge carrier diffusion length in HOIPs and the built-in field at the perovskite–electrolyte junction. The low frequency charge transfer efficiency for Pe–E device exceeds unity. This can be attributed to the modification of potential across the perovskite active layer due to ion migration at low frequency and interference from the redox reactions and formation of Helmholtz layer at the perovskite–electrolyte interface.

The general method of interpretation of the IMPS Nyquist spectra of solid-state PSC involves the association of characteristic time constants to the transport signals. Usually, the Q-plane plot consist of one or two arcs in the upper quadrant (HF region)^72–74^, though in certain cases, a third low frequency arc is observed in the lower quadrant. The HF arc is generally attributed to either electronic carrier transport within the perovskite or selective contacts or to the ionic movement within the HOIP materials^74–76^. However, Pockett et al., claimed that the series resistance and geometric capacitance of the cell is responsible for this HF arc^37^. The clear understanding of the low-frequency arc in the lower quadrant is missing. However, explanations related to slow charge recombination and transfer in PSC based on the photoelectrochemical model developed by Peter et al. is provided and charge transfer efficiencies for PSCs have been calculated^37,77^. Pockett et al. has attributed this LF IMPS feature of PSC to the relaxation of photogenerated charge carriers due to interfacial charge transport and recombination at short-circuit, in the view of similarity of PSCs with the model developed for semiconductor–electrolyte interface^37^. While the analysis based on this model is useful to a certain extent, there is a limit to the amount of information that can be extracted from this strategy. In addition, the existing interpretation of IMPS spectra for PSCs are questionable particularly with respect to the photogenerated charge carrier transport. The analysis of experimental IMPS data by modelling the electrical equivalent circuit (EEC) built from electrical elements such as resistances, capacitances and inductors like IS can provide key information about the nature of operation of the photovoltaic device and its limitations. However, the choice of the correct EEC is a difficult task since several EECs can reproduce the identical experimental spectrum. Therefore, further investigations are needed to combine the analysis of more than one small perturbation techniques to establish a universal EEC for PSCs^45^.

## Conclusion

In conclusion, we have explored the charge and ion kinetics at polymer–aqueous electrolyte and perovskite–electrolyte interface using small perturbation frequency domain techniques. It was observed that the ion migration in hybrid perovskites is highly bias dependent leading to change in the impedance and ac conductivity of the device as a function of applied bias. However, the electronic charge transport in polymer–electrolyte interface is bias independent over the entire frequency range. The spiral trajectory in Nyquist plot of intensity modulated photocurrent spectroscopy of perovskite–electrolyte interface reveals the three distinct processes going on in the device at different time scale which was overwise not possible to elucidate from impedance measurements. Hence, IMPS is a promising technique to study ion migration in HOIPs alternative to PEIS. The charge transfer to the liquid electrolyte is more efficient in Pe–E interface as compared to Po–aqE interface attributed to high carrier diffusion length in HOIPs. Ion migration induced modification in the field across the interface leads to charge transfer efficiency greater than unity at low frequency regime. Moreover, it was also confirmed that the mixed electronic–ionic interaction and multiple ion migration in hybrid perovskites is responsible for the anomalous charge transfer and recombination feature particularly at mid- and low-frequencies as depicted by the presence of low-frequency spiral feature in the IMPS Nyquist plots of perovskite-based devices. These properties would enable the application of hybrid perovskites in integrated photo-rechargeable electrochemical energy storage, water splitting, carbon capture devices and other biomedical fields.

## Methods

### Materials

Formamidinium iodide (FAI), Lead Iodide (PbI_2_), *N*,*N*-dimethylformamide (DMF, anhydrous 99.8%), poly(3,4-ethylenedioxythiophene)–poly(styrenesulfonate) (PEDOT: PSS) (1.3 wt% dispersion in H_2_O, conductive grade, Sigma-Aldrich), poly(3-hexylthiophene) (P3HT) and tetrabutylammonium perchlorate (TBAClO_4_) (for electrochemical analysis, ≥ 99.0%, Sigma-Aldrich) were purchased from Sigma-Aldrich. Potassium chloride (KCl), dichloromethane (DCM) (anhydrous), chlorobenzene (CB), isopropyl alcohol (IPA), and acetone were purchased from Sisco Research Laboratories, and all the chemicals and solvents were used as received.

### Perovskite and polymer electrode fabrication

The Indium-tin oxide (ITO) coated glass substrates were ultrasonically cleaned in soap solution followed by sonication in deionized water, acetone and isopropyl alcohol successively for 15 min each. The cleaned substrates were dried up and treated with UV-ozone for 20 min. The fabrication was performed in a controlled humidity environment with the relative humidity (RH) of < 30% and room temperature around 30–35 °C. The as received PEDOT: PSS solution was diluted in IPA by 1:1 volume ratio. The prepared PEDOT: PSS precursor solution was spin coated as the hole transport layer (HTL) on the cleaned ITO substrates at the rate of 4000 rpm for 50 s with an acceleration of 5 s under ambient conditions. The HTL coated substrates were then annealed for 20 min at 120 °C. The iodine-based perovskite material was coated using two-step spin coating technique. For coating the first layer the precursor solution of 1 M PbI_2_ in DMF was prepared and filtered through a 0.45 μm polytetrafluoroethylene (PTFE) filter. The HTL coated ITO substrates were pre-heated for 5 min at 100 °C on hot plate. The pre-heated substrates were spin-coated with the PbI_2_ precursor at 6000 rpm for 60 s with an acceleration of 5 s. The PbI_2_ coated substrates were then kept on hot plate to dry for 1 h. For coating second layer, FAI was added in isopropyl alcohol (40 mg mL^−1^). The precursor is then spin coated on the PbI_2_ coated substrates at room temperature at 6000 rpm for 60 s. The fabricated FAPbI_3_ samples were annealed on a hot plate at 160 °C for 10 min. Uniform, pinhole free, smooth and highly crystalline perovskite thin films were formed as seen from the Supplementary Fig. S1. The P3HT thin films were prepared by one-step spin coating method. The polymer precursor solution was prepared by dissolving 20 mg P3HT in 1 mL chlorobenzene. The resulting precursor solution was spin-coated on ITO substrates at 1000 rpm for 30 s with an acceleration time of 5 s in the controlled humidity chamber. The samples were then annealed at 50 °C for 5 min on the hot plate to form the uniform thin films.

### Electrochemical impedance spectroscopy

Electrochemical impedance spectroscopy (EIS) measurements were performed on Zahner Electrochemical Workstation with an active area of 0.2 cm^2^ under the xenon white light source of intensity 400 W m^−2^ at different external applied bias of 0, 0.2, 0.4, 0.6, 0.8, 1.0 V applying the ac amplitude of 20 mV in the frequency range of 100 mHz to 1 MHz. The cell set-up consists of modified photoelectrochemical cell (PECC) to hold the liquid electrolyte in two electrode system with perovskite or polymer coated ITO substrates as working electrode and platinum wire as counter electrode. The liquid electrolyte used is 0.1 M solution of tetrabutylammonium perchlorate (TBAClO_4_) in dichloromethane (DCM) for perovskite films as working electrode. The perovskite films are stable in this liquid electrolyte^23,39,78,79^. For polymer films as working electrode, 0.1 M aqueous solution of KCl was used as liquid electrolyte. The EIS data were analysed using zView software. All the measurements were carried out in the ambient air atmosphere.

### Intensity-modulated photocurrent spectroscopy

IMPS measurements were performed using Zahner CIMPS system. The measurements were carried out at short circuit potentiostatic condition with zero set voltage in the frequency range 10 kHz to 100 mHz. A white light source (1312wlr02) emitting at a wavelength of 600 nm with a spectral half width of 105 nm was used for all the measurements under DC bias of 300 W m^−2^ with an AC amplitude of 10% of the DC light intensity used. The degradation of the devices was tested under 400 W m^−2^ light illumination. A white light source (1312wlr02) emitting at a wavelength of 600 nm with a spectral half width of 105 nm was used for all the measurements The devices were kept under this light intensity for 30 min.

## Supplementary Information

Supplementary Information.

## Data Availability

All data generated or analysed during this study are included in this published article [and its supplementary information files].
