# Supplementary material for: Intensity modulated photocurrent spectroscopy to investigate hidden kinetics at hybrid perovskite–electrolyte interface
Source: Sci Rep. 2022 Aug 20;12:14212. doi: 10.1038/s41598-022-16353-6 (PMC9392765; doi:10.1038/s41598-022-16353-6)
Supplement: Supplementary file 1 — Supplementary Information. [file 41598_2022_16353_MOESM1_ESM.pdf]

Supporting Information

Intensity modulated photocurrent spectroscopy to investigate hidden kinetics at hybrid perovskite-electrolyte interface

Priya Srivastava[†], *Ramesh Kumar*[†], *Hemant Ronchiya*[†], *Monojit Bag*[†,‡,*]

† Advanced Research in Electrochemical Impedance Spectroscopy Laboratory, Indian Institute of Technology Roorkee, Roorkee 247667, India

‡ Centre of Nanotechnology, Indian Institute of Technology Roorkee, Roorkee 247667, India

*Corresponding author: monojit.bag@ph.iitr.ac.in

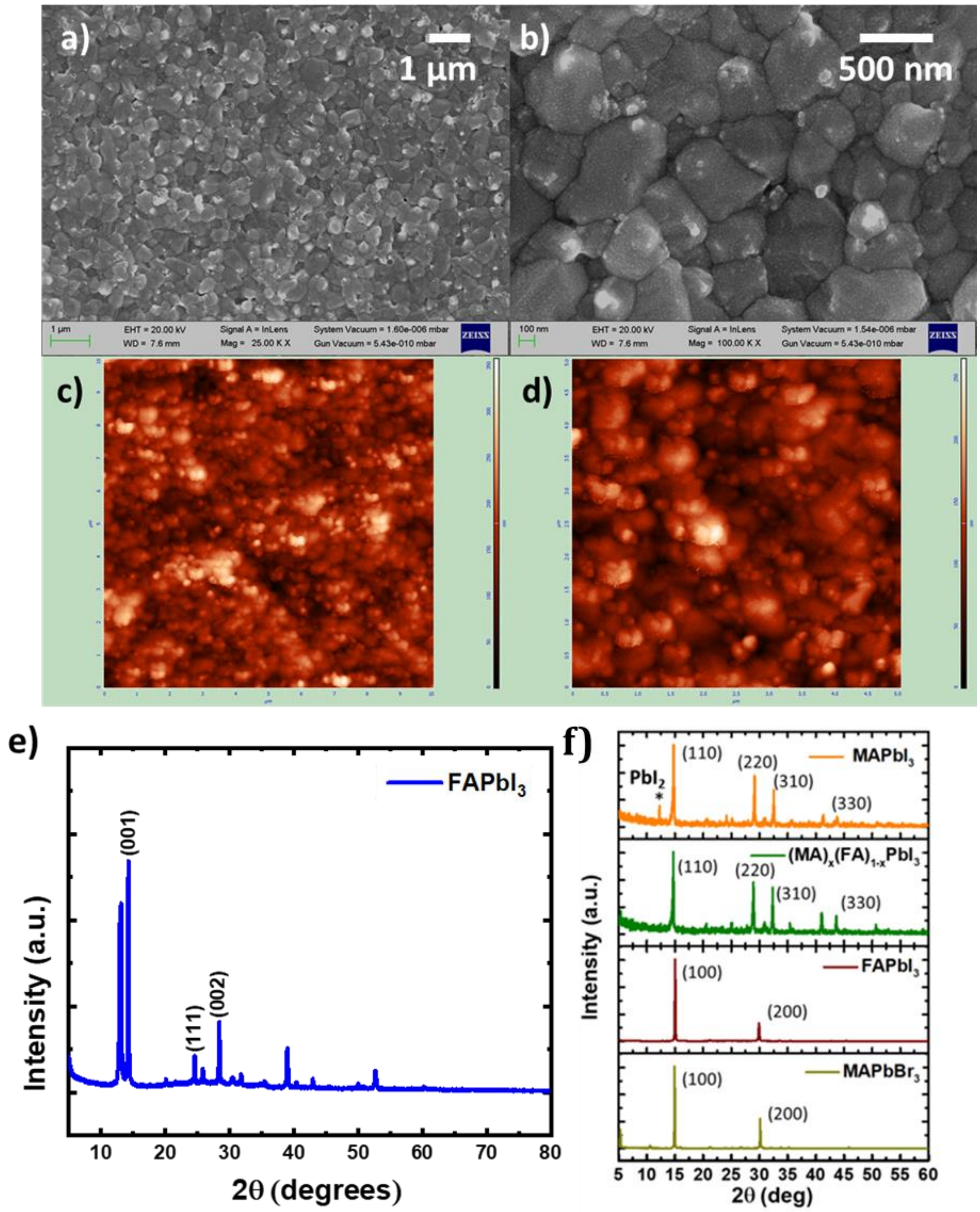

Figure S1 a, b) FESEM c, d) AFM and e, f) XRD of the ITO/PEDOT: PSS/$FAPbI_3$ thin films.

Figure S1 e shows the XRD pattern of the aged samples (after EIS measurements) to check the stability. We have observed PbI_2 secondary phase concentration in the aged samples after EIS measurements. However, we did not observe any secondary phase of PbI_2 concentration in the pristine films (Figure S1 f).[1]

Electrochemical Impedance Response

The charge carrier kinetics at perovskite-electrolyte (Pe-E) and polymer-aqueous electrolyte (Po-aqE) interface was investigated by performing the electrochemical impedance spectroscopy measurements. The impedance measurements were performed in two- electrode setup with perovskite or polymer coated ITO as working electrode and platinum as the counter electrode. The impedance measurements were performed under the illumination of intensity 400 W/m^2 in the frequency range of 1 MHz to 100 mHz at different applied dc bias of 0, 0.2, 0.4, 0.6, 0.8 and 1 V. The Nyquist and Bode plots of both are shown in Figure 1. Generally, the Nyquist plot for Pe-E and Po-aqE interface is high frequency semi-circle followed by low frequency arc. The high frequency semi-circle corresponds to the electronic charge transfer/transport and recombination at the interfaces. The absence of high-frequency semicircle here can be attributed to very fast interfacial recombination process at frequency which is out of the measured range. In Pe-E device, the low-frequency arc is associated to ion trapping and migration in the perovskite or diffusion to the electrolyte, dielectric relaxation in the perovskite active layer, electronic-ionic interaction and redox reaction at the solid-liquid junction. However, unlike perovskites, organic semiconductors such as poly(3-hexylthiophene-2,5-diyl) (P3HT) are not mixed electronic-ionic conductor. The kinetics due to the migration of ions through vacancies and interaction with electronic charge carriers are absent in this organic semiconductor. For the P3HT-electrolyte devices the high frequency kinetics is mostly because of the electronic charge carrier recombination, transfer and trapping at the solid-liquid interface whereas the low frequency kinetics can be attributed to the photo-oxidation along with enhanced percolation of electrolyte through polymer bulk.[2]

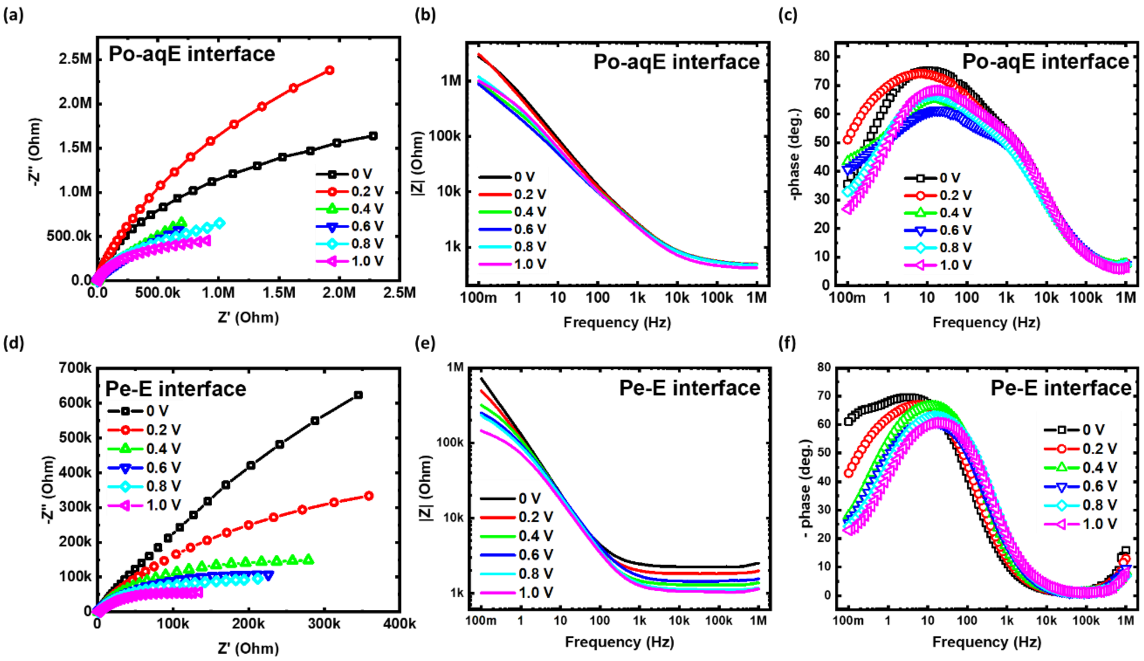

Figure S2. Nyquist and Bode plots for Po-aqE interface (a, b, c) and Pe-E interface (d, e, f) respectively.

It can be noted that the impedance at Pe-E interface varies gradually with the applied bias, whereas it follows different behavior in Po-aqE interface. In case of polymer-electrolyte interface, the impedance is very high (~ MΩ) for lower applied bias and reduces suddenly after 0.2 V applied bias. Even after 0.2 V applied bias, it is not much bias dependent. This can be speculated to the diffusion limited transport of electronic charge carriers at short-circuit or low bias (0 – 0.2 V) conditions which turns to drift current only after 0.4 V applied bias. This will be explained in more detail in the next section where the IMPS Nyquist plot for Po-aqE device shows the diffusion controlled anodic behavior under short-circuit condition and flips to cathodic on application of external field. In contrast, the bias dependent and comparatively low impedance in Pe-E

devices can be attributed to the electronic-ionic interaction under the combined effect of built-in field, photogenerated potential and external applied bias.[3,4]

To get more insight into the charge transfer and accumulation at these two types of interfaces real and imaginary capacitance were calculated from the measured impedance according to the following equations:

$$C_R(\omega) = -\frac{Im(Z)}{\omega|Z|^2} \; ; \; C_{Im}(\omega) = \frac{Re(Z)}{\omega|Z|^2} \qquad 5$$

where ω is the applied ac frequency, |Z| is the modulus of measured impedance Z, Re(Z) and Im(Z) are real and imaginary part of the impedance. Figure 2 (a, b, c, d) shows the frequency dependance of the real and imaginary capacitance for the Pe-E and Po-aqE devices. Real capacitance is related to the energy storage whereas imaginary capacitance is associated with the dielectric loss relating to dissipation of energy. In the high frequency region, C_{Im} vs. f plot shows ~1/f dependance and hence follows Jonscher's power law (JPL). At mid-frequency regime, frequency independent plateau leading to nearly constant dielectric loss (NCL) is present in Pe-E device. However, in Po-aqE device slight NCL region can be seen only for low applied bias (0 and 0.2 V). Moreover, unlike Pe-E interface the capacitance at low frequency (< 100 Hz) increases with bias till 0.2 or 0.3 V and saturates thereafter in Po-aqE interface. This is in agreement with the variation of Helmholtz capacitance with bias observed by Tullii et. al.[2] The high capacitance at very low frequency is due to a pseudo-capacitance contribution originated from the reversible intercalation of redox ions at the polymer-electrolyte interface, enhanced by the occurrence of faradaic processes.[2] However, in Pe-E interface correlated ion transport and accumulation is responsible for this.[1]

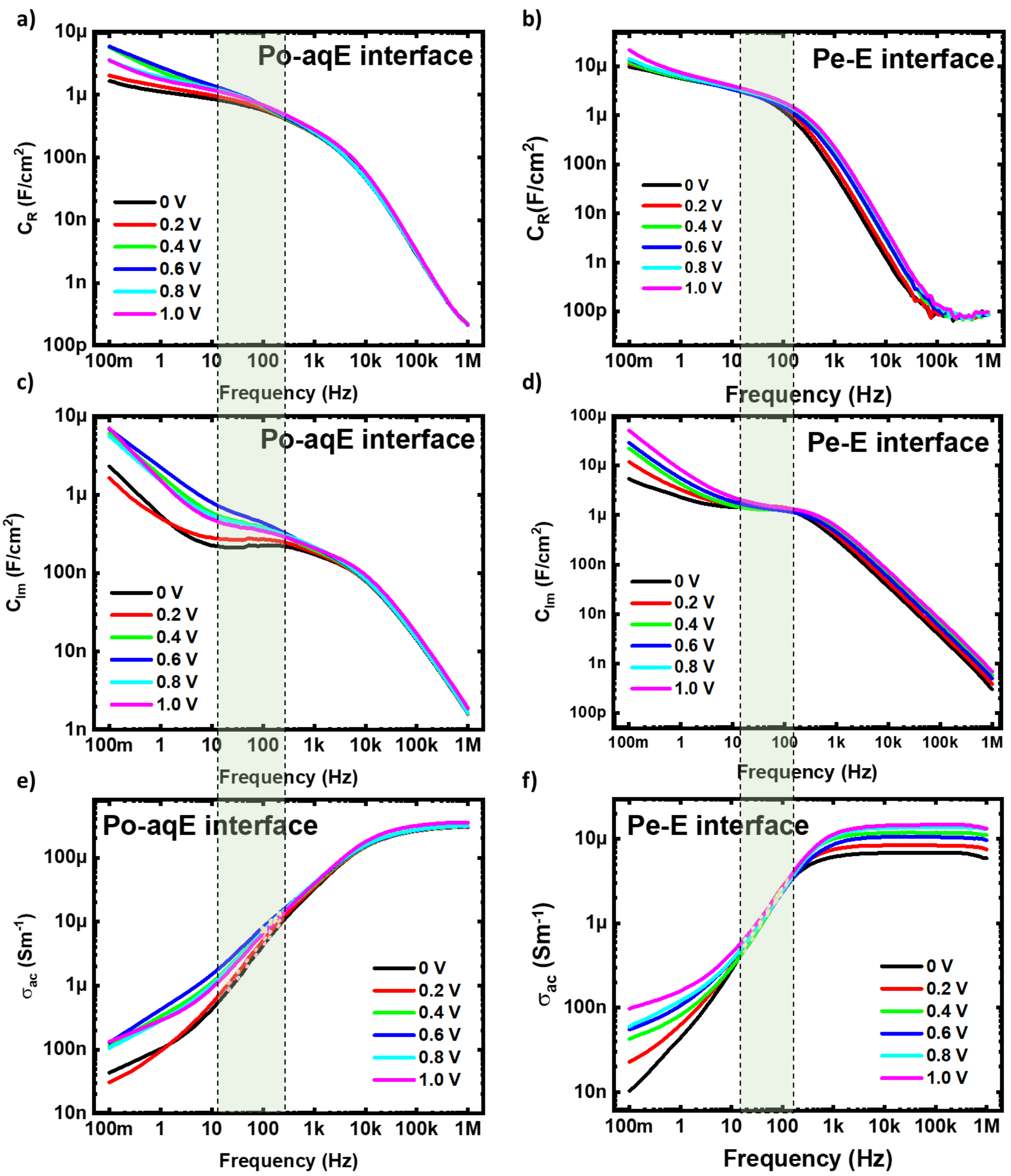

Figure S3. Real capacitance (C_R), Imaginary capacitance (C_{Im}), and ac conductivity (σ_{ac}) for Po-aqE (a, c, e) and Pe-E (b, d, f) interface respectively.

The a.c. conductivity is determined using the JPL equation $\sigma_{a.c} = \epsilon_0 \epsilon'' \omega$ from impedance spectroscopy data according to the previous reports.[5,6] The a.c. conductivity vs. frequency feature for both Po-aqE and Pe-E shows similar nature of high frequency plateau followed by decrease in conductivity with decrease in frequency (Figure 2 e, f). The ac

conductivity in Pe-E device is bias dependent both at high and low frequency regime with a NCL feature at mid-frequency. However, in Po-aqE interface it is bias independent over the whole range of applied ac frequency. For frequencies < 100 Hz, a small difference can be seen on changing the bias from 0.2 to 0.4 V. This behavior could be attributed to the absence of bias dependent migration of ions in P3HT. There is a possibility of contribution from the ions in the electrolyte though at very low frequency. It is reported that, the aqueous electrolyte polarizes the polymer outermost layer and localizes the photogenerated charge carriers together with p-(photo) doping by dissolved oxygen.[7] Under illumination, the Po-aqE interface is negatively charged and attracts positive ions from the electrolyte solution, perturbing the ion-distribution in aqueous electrolyte. This process might be assisted by the applied external bias to the interface leading to sudden change after V_{app} = 0.2 V.

Methods

Intensity-Modulated Photovoltage Spectroscopy

IMVS measurements were performed using Zahner CIMPS system. The measurements were carried out at open circuit condition in the frequency range 10 kHz to 100 mHz. A white light source (1312wlr02) emitting at a wavelength of 600 nm with a spectral half width of 105 nm was used for all the measurements under DC bias of 300 W/m^2 with an AC amplitude of 10% of the DC light intensity used.

Transient Photovoltage Measurement

The photovoltage measurements were performed in the two-electrode electrochemical setup. A white light LED was used as light source regulated by a function generator. The photovoltage spectrum was recorded by a digital oscilloscope. Crocodile clips were used to connect the electrodes with the external circuit.

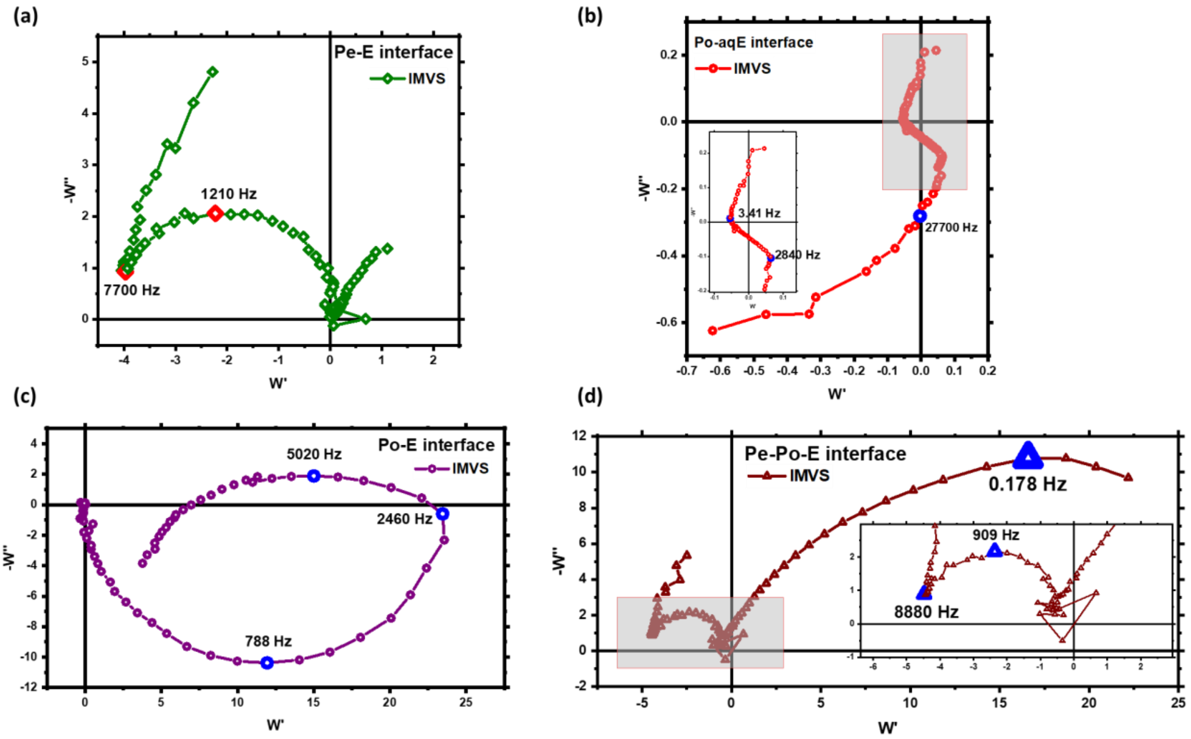

Figure S4. IMVS Nyquist plot of a) Pe-E (perovskite-electrolyte interface), b) Po-aqE (polymer-aqueous electrolyte interface), c) Po-E (polymer-electrolyte interface) and d) Pe-Po-E (perovskite-polymer-electrolyte interface).

Transient Photovoltage Spectroscopy

Small-perturbation transient photovoltage (TPV) decay response of perovskite-electrolyte (Pe-E) and polymer-aqueous electrolyte (Po-aqE) interface was measured at different illumination frequency as an assay of charge-carrier recombination kinetics at the interface. The TPV measurements are desirable to quantify recombination times and charge extraction rate. The transient photovoltage is given as

$$\Delta V = \Delta V_0 e^{-\frac{t}{\tau}} \quad (S4)$$

Here, ΔV (V) is photovoltage response, t is the time, and τ is the recombination life time. The recombination lifetime calculated from typical transient photovoltage responses of both devices are shown in the Figure S5. It can be noted that the recombination life time of perovskite-electrolyte and polymer-electrolyte devices are in order of 0.1 ms and 0.01 µs, respectively. The higher life time in the perovskite materials might be related to halide ion migration in the perovskite bulk, while as in polymer-electrolyte the recombination life time is related to the electronic charge carrier.

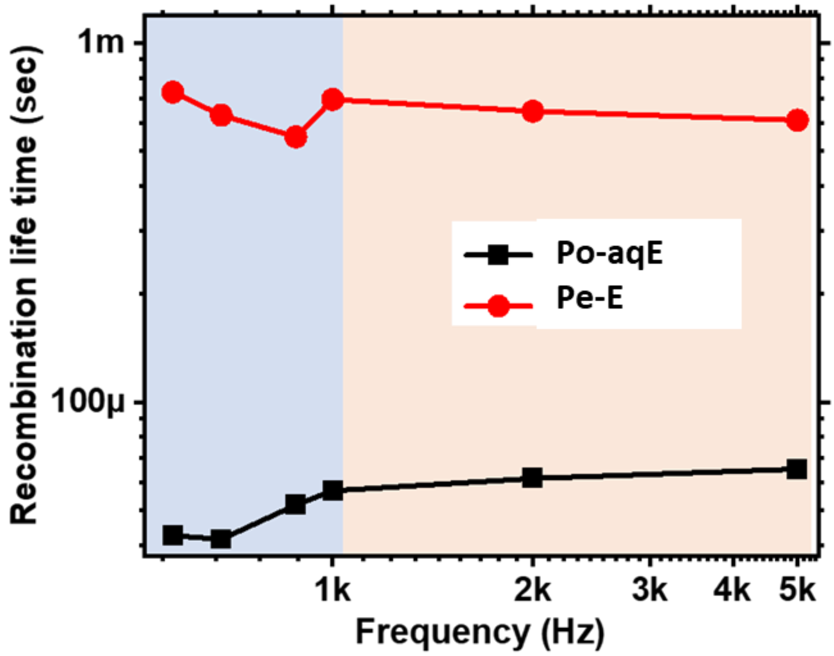

Figure S5. Recombination lifetime at different frequency for Po-aqE and Pe-E interface from TPV.

References:

1. Srivastava, P., Kumar, R. & Bag, M. Discerning the Role of an A-Site Cation and X-Site Anion for Ion Conductivity Tuning in Hybrid Perovskites by Photoelectrochemical Impedance Spectroscopy. *J. Phys. Chem. C* **0**, 211–222 (2021).

2. Tullii, G. *et al.* Bimodal functioning of a mesoporous, light sensitive polymer/electrolyte interface. *Org. Electron.* **46**, 88–98 (2017).

3. Smith, E. C. *et al.* Interplay between Ion Transport, Applied Bias, and Degradation under Illumination in Hybrid Perovskite p-i-n Devices. *J. Phys. Chem. C* **122**, 13986–13994 (2018).

4. Srivastava, P. & Bag, M. Elucidating Tuneable Ambipolar Charge Transport and Field Induced Bleaching at CH3NH3PbI3/Electrolyte Interface. *Phys. Chem. Chem.*

Phys. (2020) doi:10.1039/d0cp00682c.

5. Kumar, R., Srivastava, P., Bag, M., Kumar, R. & Srivastava, P. Role of A-Site Cation and X-Site Halide Interactions in Mixed-Cation Mixed-Halide Perovskites for Determining Anomalously High Ideality Factor and the Super-linear Power Law in AC Ionic Conductivity at Operating Temperature. *ACS Appl. Electron. Mater.* **12**, 4087–4098 (2020).

6. Srivastava, P., Kumar, R. & Bag, M. The Curious Case of Ion Migration in Solid-state and Liquid Electrolyte-based Perovskite Devices: Unveiling the Role of Charge Accumulation and Extraction at the Interfaces. *Phys. Chem. Chem. Phys.* **23**, 10936–10945 (2021).

7. Mosconi, E. *et al.* Surface Polarization Drives Photoinduced Charge Separation at the P3HT/Water Interface. *ACS Energy Lett.* **1**, 454–463 (2016).
